# Supplementary material for: Microbiome Responses to Fecal Microbiota Transplantation in Cats with Chronic Digestive Issues
Source: Vet Sci. 2023 Sep 6;10(9):561. doi: 10.3390/vetsci10090561 (PMC10537086; doi:10.3390/vetsci10090561)
Supplement: Supplementary file 1 [file vetsci-10-00561-s001.zip › vetsci-2517899-Supplementary_Figures.pdf]

SUPPLEMENTARY FIGURES FOR:

Microbiome responses to fecal microbiota transplantation in cats with chronic digestive issues

Connie A. Rojas[1,2], Zhandra Entrolezo[3], Jessica Jarett[3], Guillaume Jospin[3], Dawn Kingsbury[3], Alex Martin[3], Jonathan A. Eisen[1,2], Holly H. Ganz[3]

[1] Genome Center, University of California – Davis, Davis, California, USA
[2] Department of Evolution and Ecology, University of California – Davis, Davis, California, USA
[3] AnimalBiome, Oakland, CA, USA

Fig S1. Fecal microbiome composition before and after FMT for the forty-six FMT recipients. Plots showing the relative frequencies of 16S rRNA gene sequences assigned to bacterial genera with mean relative abundances > 1.65%, while all other genera are clumped into an "Other" category.

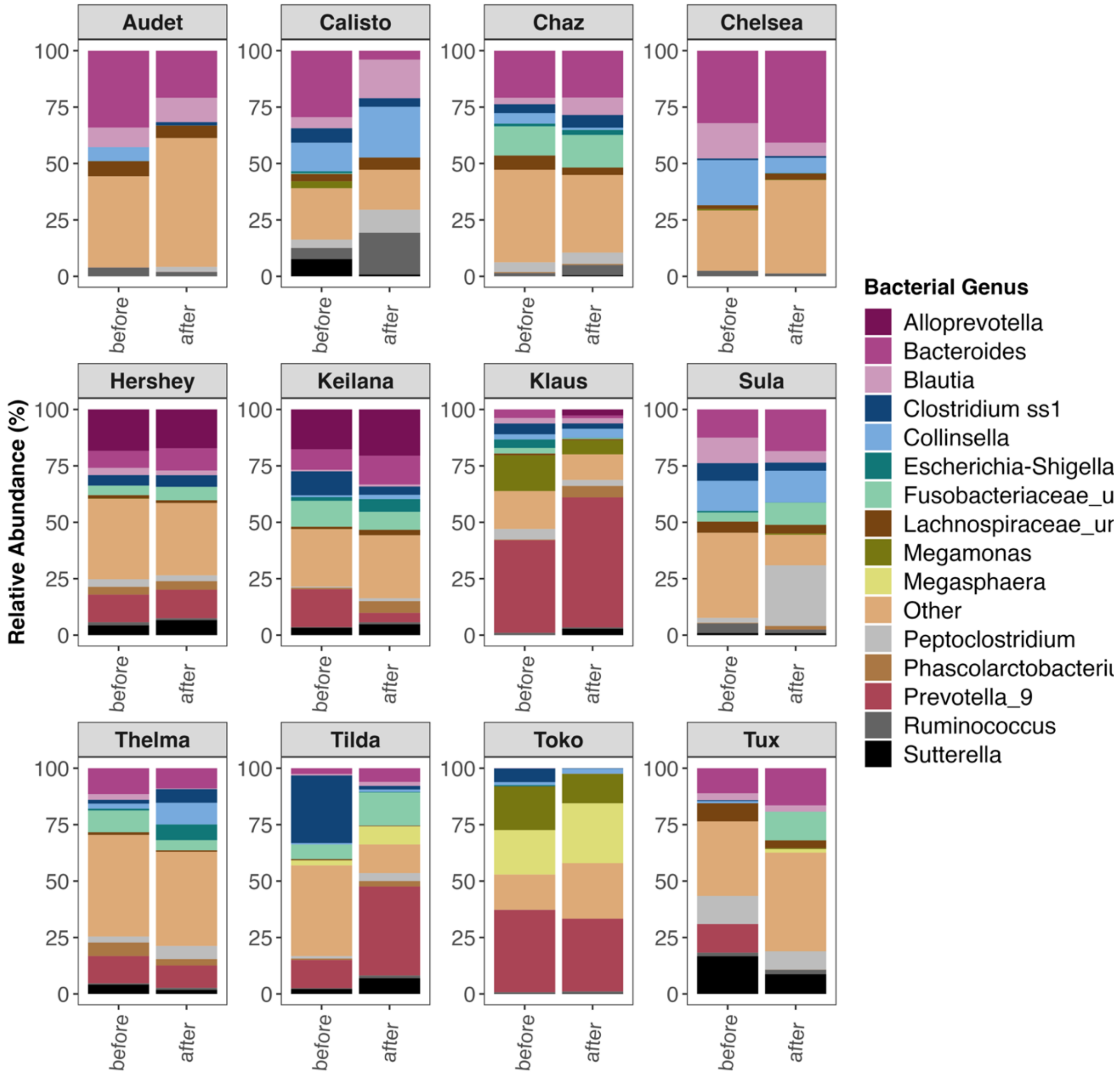

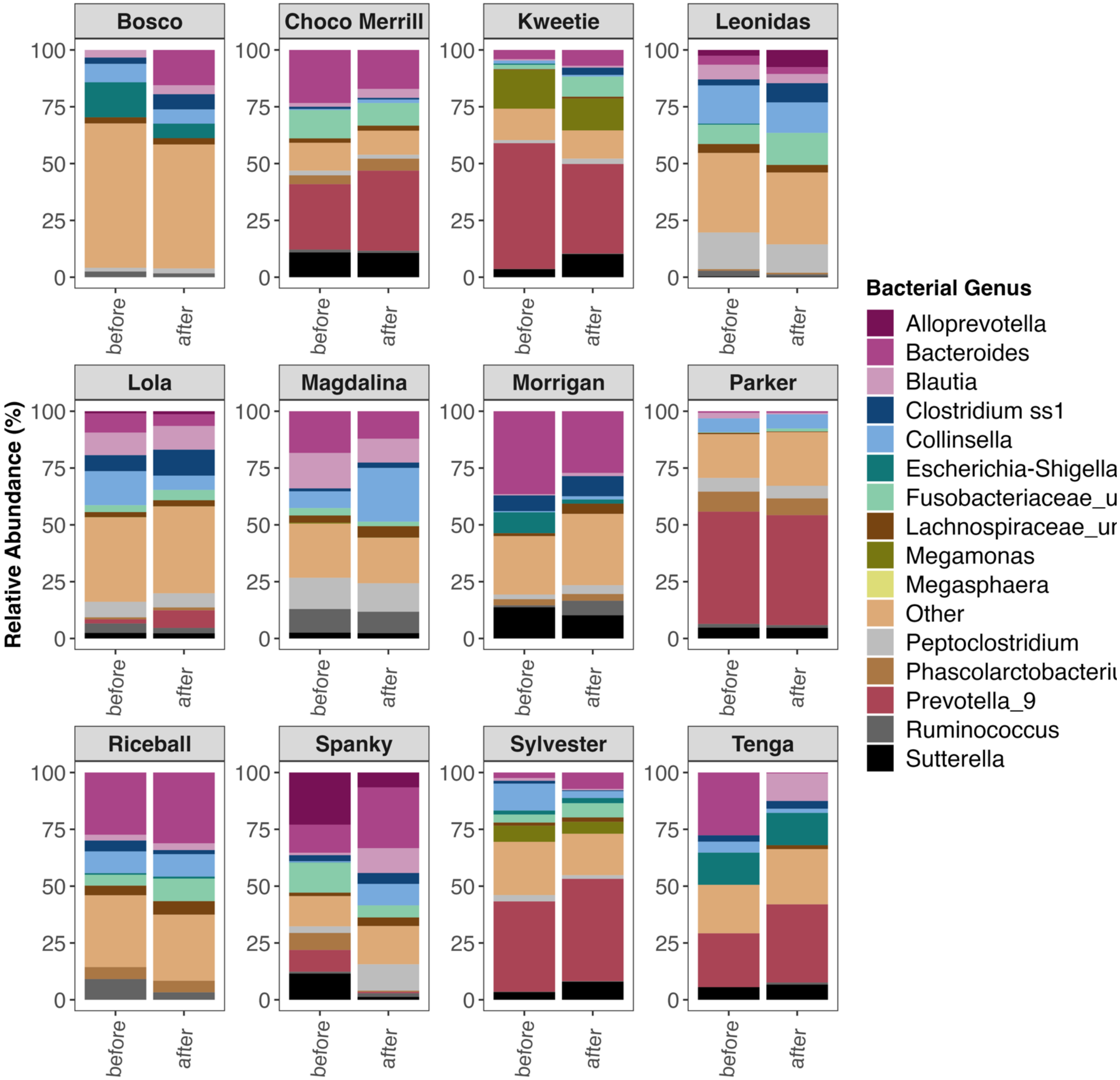

Bosco
Choco Merrill
Kweetie
Leonidas
Lola
Magdalina
Morrigan
Parker
Riceball
Spanky
Sylvester
Tenga
Relative Abundance (%)
100
75
50
25
0
before
after
Bacterial Genus
Alloprevotella
Bacteroides
Blautia
Clostridium ss1
Collinsella
Escherichia-Shigella
Fusobacteriaceae_u
Lachnospiraceae_ur
Megamonas
Megasphaera
Other
Peptoclostridium
Phascolarctobacteriu
Prevotella_9
Ruminococcus
Sutterella

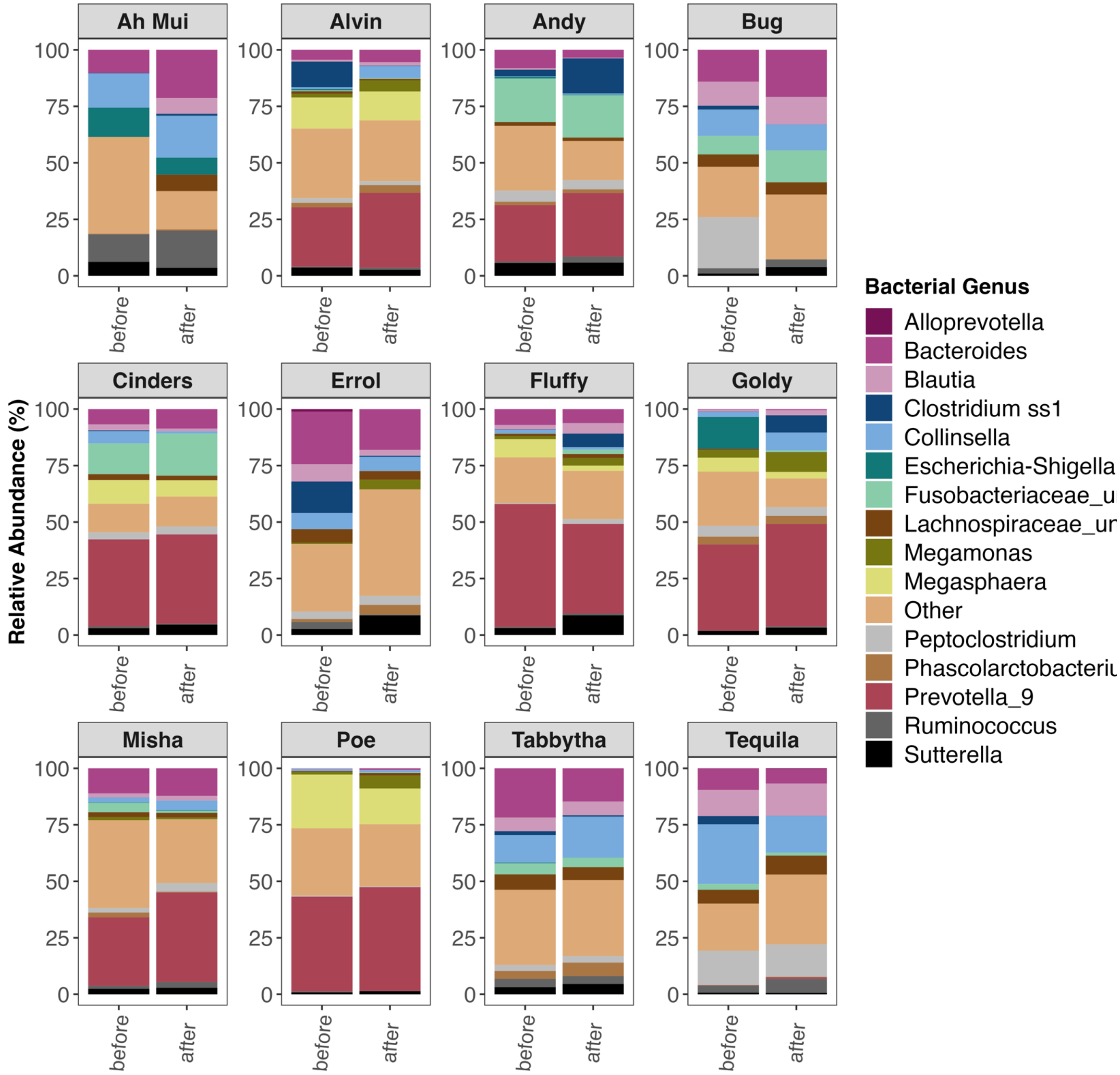

Ah Mui
Alvin
Andy
Bug
Cinders
Errol
Fluffy
Goldy
Misha
Poe
Tabbytha
Tequila
Relative Abundance (%)
100
75
50
25
0
before
after
Bacterial Genus
Alloprevotella
Bacteroides
Blautia
Clostridium ss1
Collinsella
Escherichia-Shigella
Megamonas
Megasphaera
Other
Peptoclostridium
Prevotella_9
Ruminococcus
Sutterella

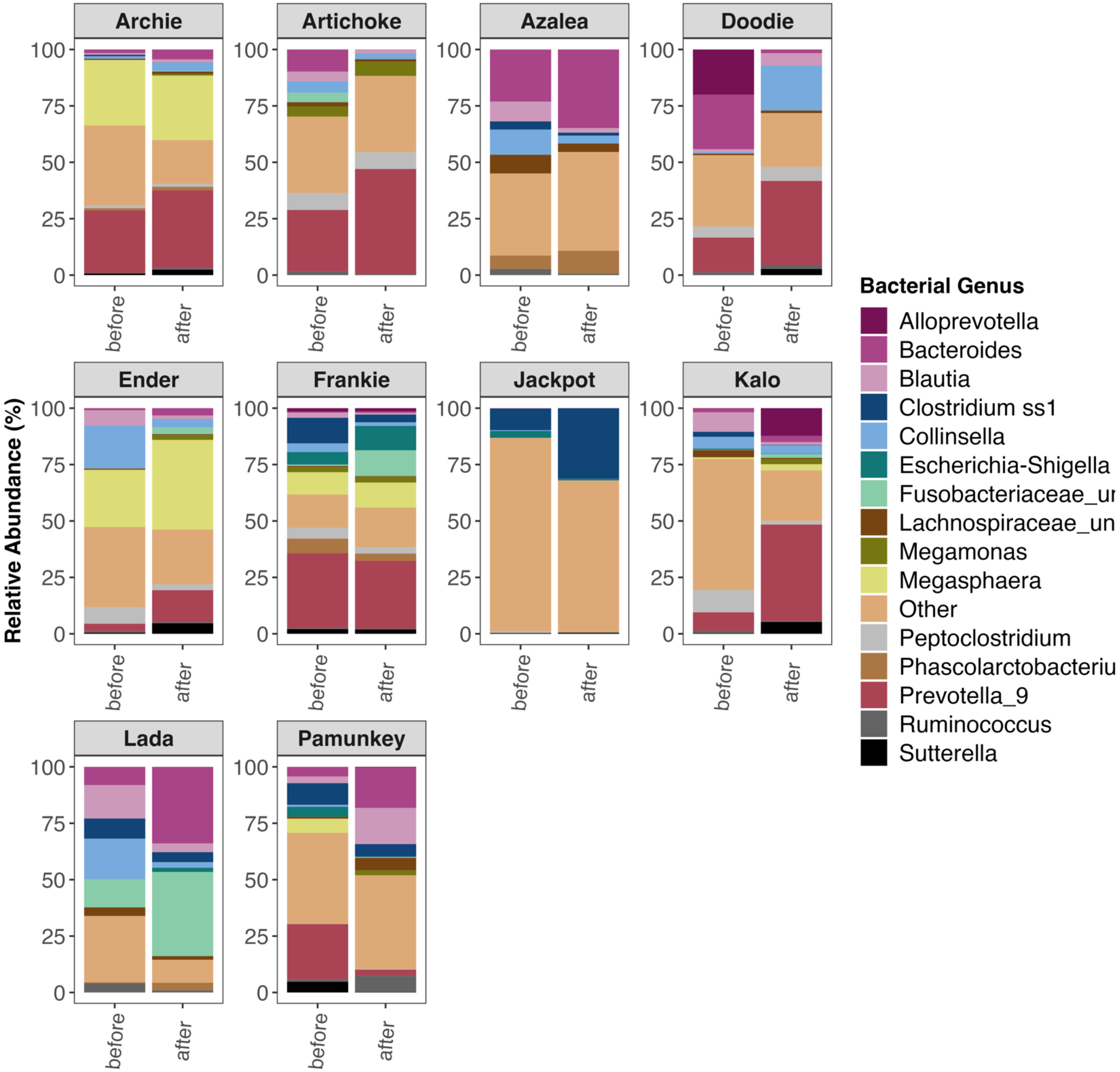
Archie
Artichoke
Azalea
Doodie
Ender
Frankie
Jackpot
Kalo
Lada
Pamunkey
Relative Abundance (%)
0
25
50
75
100
before
after
Bacterial Genus
Alloprevotella
Bacteroides
Blautia
Clostridium ss1
Collinsella
Escherichia-Shigella
Fusobacteriaceae_ur
Lachnospiraceae_un
Megamonas
Megasphaera
Other
Peptoclostridium
Phascolarctobacteriu
Prevotella_9
Ruminococcus
Sutterella

Fig S2. Posthoc testing of bacterial genera that were found to significantly correlate with host predictors according to a linear model. A-D) Posthoc testing of bacterial relative abundances ~ clinical symptoms, E-G) Posthoc testing of bacterial relative abundances ~ response to FMT, H) Posthoc testing of bacterial relative abundances ~ prior antibiotic use, and I) Posthoc testing of bacterial relative abundances ~ Dry kibble consumption. Tukey contrasts were used.

A) GLM Δ Relative Abundance of Clostridium ~ Clinical signs

```
Linear Hypotheses:
                              Estimate Std. Error z value Pr(>|z|)
Diarrhea - Constipation == 0   -0.8888     2.9916  -0.297   0.9905
VomConstip - Constipation == 0 -4.6916     4.2027  -1.116   0.6709
VomDiarr - Constipation == 0    4.6072     3.1022   1.485   0.4362
VomConstip - Diarrhea == 0     -3.8027     3.7594  -1.012   0.7352
VomDiarr - Diarrhea == 0        5.4960     2.3325   2.356   0.0816 .
VomDiarr - VomConstip == 0      9.2987     3.9539   2.352   0.0825 .
---
Signif. codes:  0 ‘***’ 0.001 ‘**’ 0.01 ‘*’ 0.05 ‘.’ 0.1 ‘ ’ 1
(Adjusted p values reported -- single-step method)
```

B) GLM Δ Relative Abundance of Collinsella ~ Clinical signs

```
Linear Hypotheses:
                              Estimate Std. Error z value Pr(>|z|)
Diarrhea - Constipation == 0    -4.349      2.899  -1.500   0.4271
VomConstip - Constipation == 0  -7.245      4.072  -1.779   0.2743
VomDiarr - Constipation == 0    -8.457      3.006  -2.813   0.0237 *
VomConstip - Diarrhea == 0      -2.896      3.643  -0.795   0.8519
VomDiarr - Diarrhea == 0        -4.109      2.260  -1.818   0.2562
VomDiarr - VomConstip == 0      -1.212      3.831  -0.316   0.9886
---
Signif. codes:  0 ‘***’ 0.001 ‘**’ 0.01 ‘*’ 0.05 ‘.’ 0.1 ‘ ’ 1
(Adjusted p values reported -- single-step method)
```

C) GLM Δ Relative Abundance of Negativibacillus ~ Clinical signs

```
Linear Hypotheses:
                               Estimate Std. Error z value Pr(>|z|)
Diarrhea - Constipation == 0    -0.4215     0.7521  -0.560  0.94157
VomConstip - Constipation == 0   2.4778     1.0565   2.345  0.08423 .
VomDiarr - Constipation == 0    -0.8434     0.7799  -1.082  0.69261
VomConstip - Diarrhea == 0       2.8993     0.9451   3.068  0.01104 *
VomDiarr - Diarrhea == 0        -0.4219     0.5864  -0.720  0.88547
VomDiarr - VomConstip == 0      -3.3212     0.9940  -3.341  0.00481 **
---
Signif. codes:  0 '***' 0.001 '**' 0.01 '*' 0.05 '.' 0.1 ' ' 1
(Adjusted p values reported -- single-step method)
```

D) GLM Δ Relative Abundance of Subdoligranulum ~ Clinical signs

```
Linear Hypotheses:
                               Estimate Std. Error z value Pr(>|z|)
Diarrhea - Constipation == 0     0.3531     0.6903   0.511  0.95471
VomConstip - Constipation == 0  -2.2919     0.9698  -2.363  0.08036 .
VomDiarr - Constipation == 0     0.6407     0.7159   0.895  0.80131
VomConstip - Diarrhea == 0      -2.6450     0.8675  -3.049  0.01167 *
VomDiarr - Diarrhea == 0         0.2876     0.5382   0.534  0.94883
VomDiarr - VomConstip == 0       2.9326     0.9124   3.214  0.00687 **
---
Signif. codes:  0 '***' 0.001 '**' 0.01 '*' 0.05 '.' 0.1 ' ' 1
(Adjusted p values reported -- single-step method)
```

E) GLM Δ Relative Abundance of Butyricoccus ~ Response to FMT

```
Linear Hypotheses:
                                Estimate Std. Error z value Pr(>|z|)
Responder - Non-Responder == 0 -0.10298    0.05364   -1.92   0.0549 .
---
Signif. codes:  0 '***' 0.001 '**' 0.01 '*' 0.05 '.' 0.1 ' ' 1
(Adjusted p values reported -- single-step method)
```

F) GLM Δ Relative Abundance of Megamonas ~ Response to FMT

```
Linear Hypotheses:
                               Estimate Std. Error z value Pr(>|z|)
Responder - Non-Responder == 0    2.108      0.782   2.696  0.00702 **
---
Signif. codes:  0 ‘***’ 0.001 ‘**’ 0.01 ‘*’ 0.05 ‘.’ 0.1 ‘ ’ 1
(Adjusted p values reported -- single-step method)
```

G) GLM Δ Relative Abundance of Peptococcus ~ Response to FMT

```
Linear Hypotheses:
                               Estimate Std. Error z value Pr(>|z|)
Responder - Non-Responder == 0  -0.3941     0.1572  -2.506   0.0122 *
---
Signif. codes:  0 ‘***’ 0.001 ‘**’ 0.01 ‘*’ 0.05 ‘.’ 0.1 ‘ ’ 1
(Adjusted p values reported -- single-step method)
```

H) GLM Δ Relative Abundance of Ruminococcus ~ Response to FMT

```
Linear Hypotheses:
                               Estimate Std. Error z value Pr(>|z|)
Responder - Non-Responder == 0  -2.1334     0.9945  -2.145   0.0319 *
---
Signif. codes:  0 ‘***’ 0.001 ‘**’ 0.01 ‘*’ 0.05 ‘.’ 0.1 ‘ ’ 1
(Adjusted p values reported -- single-step method)
```

I) GLM Δ Relative Abundance of Negativibacillus ~ Prior antibiotic use

```
Linear Hypotheses:
             Estimate Std. Error z value Pr(>|z|)
Yes - No == 0  -1.0947     0.5276  -2.075    0.038 *
---
Signif. codes:  0 ‘***’ 0.001 ‘**’ 0.01 ‘*’ 0.05 ‘.’ 0.1 ‘ ’ 1
(Adjusted p values reported -- single-step method)
```

J) GLM Δ Relative Abundance of Peptoclostridium ~ diet (dry kibble consumption)

```
Linear Hypotheses:
             Estimate Std. Error z value Pr(>|z|)
Yes - No == 0   -3.470      1.834  -1.892   0.0585 .
---
Signif. codes:  0 ‘***’ 0.001 ‘**’ 0.01 ‘*’ 0.05 ‘.’ 0.1 ‘ ’ 1
(Adjusted p values reported -- single-step method)
```

K) GLM Δ Relative Abundance of Subdoligranulum ~ diet (dry kibble consumption)

```
Linear Hypotheses:
             Estimate Std. Error z value Pr(>|z|)
Yes - No == 0  -1.1032     0.5046  -2.186   0.0288 *
---
Signif. codes:  0 ‘***’ 0.001 ‘**’ 0.01 ‘*’ 0.05 ‘.’ 0.1 ‘ ’ 1
(Adjusted p values reported -- single-step method)
```

L) GLM Δ Relative Abundance of Desulfovibrio ~ clinical signs

```
Linear Hypotheses:
                               Estimate Std. Error z value Pr(>|z|)
Diarrhea - Constipation == 0   -0.13716    0.36311  -0.378  0.98091
VomConstip - Constipation == 0  1.43156    0.51011   2.806  0.02418 *
VomDiarr - Constipation == 0   -0.18558    0.37654  -0.493  0.95920
VomConstip - Diarrhea == 0      1.56872    0.45631   3.438  0.00315 **
VomDiarr - Diarrhea == 0       -0.04842    0.28311  -0.171  0.99815
VomDiarr - VomConstip == 0     -1.61714    0.47991  -3.370  0.00384 **
---
Signif. codes:  0 ‘***’ 0.001 ‘**’ 0.01 ‘*’ 0.05 ‘.’ 0.1 ‘ ’ 1
(Adjusted p values reported -- single-step method)
```

M) GLM Δ Relative Abundance of Veillonella ~ response to FMT

```
Linear Hypotheses:
                                Estimate Std. Error z value Pr(>|z|)
Responder - Non-Responder == 0 -0.013494   0.007379  -1.829   0.0675 .
---
Signif. codes:  0 ‘***’ 0.001 ‘**’ 0.01 ‘*’ 0.05 ‘.’ 0.1 ‘ ’ 1
(Adjusted p values reported -- single-step method)
```

N) GLM Δ Relative Abundance of Desulfovibrio ~ prior antibiotic use

```
Linear Hypotheses:
              Estimate Std. Error z value Pr(>|z|)
Yes - No == 0  -0.4849     0.2548  -1.903    0.057 .
---
Signif. codes:  0 ‘***’ 0.001 ‘**’ 0.01 ‘*’ 0.05 ‘.’ 0.1 ‘ ’ 1
(Adjusted p values reported -- single-step method)
```

O) GLM Δ Relative Abundance of Escherichia ~ prior antibiotic use

```
Linear Hypotheses:
              Estimate Std. Error z value Pr(>|z|)
Yes - No == 0   -2.025      1.027  -1.972   0.0486 *
---
Signif. codes:  0 ‘***’ 0.001 ‘**’ 0.01 ‘*’ 0.05 ‘.’ 0.1 ‘ ’ 1
(Adjusted p values reported -- single-step method)
```
